# Antibioflm effects of extracellular matrix degradative agents on the biofilm of different strains of multi-drug resistant *Corynebacterium striatum*

**DOI:** 10.1186/s12941-022-00546-y

**Published:** 2022-11-25

**Authors:** Juan Wen, Zhaohui Wang, Xiaoli Du, Roushan Liu, Junrui Wang

**Affiliations:** 1 Department of Laboratory Medicine, Affiliated Hospital of Inner Mongolian Medical University, Hohhot, 010050 People’s Republic of China; 2 Department of Pharmacy, Affiliated Hospital of Inner Mongolian Medical University, 010050 Hohhot, People’s Republic of China; 3 National Institute for Communicable Disease Control and Prevention, Chinese Center for Disease Control and Prevention, Beijing, 102206 People’s Republic of China

**Keywords:** *Corynebacterium striatum*, Biofilm formation, Multi-drug resistance, Extracellular proteins, Exopolysaccharides, eDNA

## Abstract

**Background:**

*Corynebacterium striatum* is a microorganism with an excellent capacity for biofilm production and thus has been correlated with nosocomial transmission and invasive infections. However, little is known about the mechanism of biofilm formation of this commensal pathogen. In this study, we aimed to investigate the biofilm formation abilities of multidrug-resistant *Corynebacterium striatum* clinical isolates and the roles of extracellular proteins, exopolysaccharides and extracellular DNA in mediating more robust biofilm formation by the isolates of *C. striatum*.

**Methods:**

*C. striatum* isolates were identified using VITEK-2 ANC card, matrix-assisted laser desorption/ionization-time of flight mass spectrometry and 16S rRNA sequencing. The antibiotic susceptibility test was performed using the broth microdilution method. The distribution of *spaDEF* genes among *C. striatum* isolates was detected by polymerase chain reaction, and pulsed-field gel electrophoresis typing was employed to analyze the genotypes of the isolates. Crystal violet staining and scanning electron microscopy techniques were used to detect biofilm production by *C. striatum* isolates. Biofilm degradation assay was performed to observe the effects of extracellular matrix degradative agents (proteinase K, dispersin B, and DNase I) on *C. striatum* biofilms.

**Results:**

Twenty-seven *C. striatum* isolates were enrolled in the study, and the resistance rates were the highest (100%, 27/27) against penicillin and ceftriaxone. Approximately 96.3% (26/27) *C. striatum* isolates were resistant to at least three different types of antimicrobial agents tested. All isolates were confirmed to be biofilm producers, and 74.07% (20/27) isolates presented moderate to strong biofilm production abilities. P7 genotype (44.4%, 12/27) was identified to as the predominant genotype, and all of isolates belonging to this genotype were multidrug-resistant and had stronger biofilm-forming abilities. Most *C. striatum* isolates (74.07%, 20/27) carry *spaD*, *spaE,* and *spaF* genes, which encode spa-type pili. However, the correlation between the expression of spa-type genes and the biofilm production abilities of the *C. striatum* isolates was not found. The biofilms of 80% (8/10), 90% (9/10), and 100% (10/10) *C. striatum* isolates with moderate to strong biofilm production abilities were significantly eliminated upon the treatment of dispersin B (20 μg/mL), DNase I (20 μg/mL), and proteinase K (20 μg/mL) (*p* < 0.05), respectively. For the combination groups with two kinds of biofilm-degradative agents, the combination of 20 μg/mL proteinase K/dispersin B showed the strongest biofilm-eliminating effects, when the biofilms of 90% (9/10) *C. striatum* isolates degraded more than 50%.

**Conclusions:**

The *C. striatum* isolates that belonged to the predominant genotype showed a multidrug resistance (MDR) phenotype and strong biofilm formation abilities. Extracellular matrix seems to be an essential determinant in mediating biofilm formation of MDR *C. striatum,* since extracellular matrix degradative agents (proteinase K, dispersin B and DNase I) showed strong biofilm-eliminating effects toward multidrug-resistant *C. striatum* isolates. The findings of this study highlight new ideas/directions to explore the whole nature of biofilm formation of *C. striatum* and the function of extracellular matrix in this process.

**Supplementary Information:**

The online version contains supplementary material available at 10.1186/s12941-022-00546-y.

## Background

*Corynebacterium striatum* is a gram-positive, rod-shaped bacterium, opportunistic bacterial pathogen that has caused several types of invasive infections in the last two decades [1]. *C. striatum* clinical isolates often show a high rate of multidrug resistance [2], and its predominant have stronger biofilm formation ability, which correlates with its widespread transmission within the hospital environment [3, 4]. Unfortunately, little is known about the actual mechanism of biofilm formation of this pathogen.

Some recent reports revealed that production of biofilm was correlated with pathogenicity potential of *C. striatum*, especially for some kinds of chronic infections, including lower respiratory tract infections, catheter-related infections, and meningitis [5]. *C. striatum* can produce biofilms on different types of abiotic surfaces, which may contribute to the enhancement of its virulence and invasion ability. For some well-known bacterial pathogens, the expression of bacterial surface structure and production of extracellular matrix were recognized to be two different kinds of crucial mechanisms contributing to biofilm formation. A recent report revealed that *spa* genes, encoding essential cellular surface structure pili, were highly expressed among *C. striatum* clinical isolates and were thought to correlate with cellular adhesion and biofilm production of *C. striatum* [6]. Complex extracellular matrix correlated with biofilm formation in bacterial pathogens and is mainly composed of exopolysaccharides (EPS), proteins and extracellular DNA(eDNA) [7–9], and corresponding degradative agents (protein degradative enzymes, polysaccharide poly-N-acetyl glucosamine degradative enzymes and eDNA degradative enzymes) usually showed significant biofilm eradicating effects [8, 9]. To date, no study reported the role of extracellular matrix degradative agents in the biofilm formation process in *C. striatum*.

Hence, this study aimed to explore the biofilm formation abilities of *C. striatum* clinical isolates and the roles of pili or anti-biofilm activities of extracellular matrix degradative agents in the regulating biofilm formation.

## Methods

### Identification of *C. striatum* strains

*C. striatum* strains were isolated from the patients admitted to the Affiliated Hospital of Inner Mongolia Medical University from January 2012 to July 2021. All isolates were identified by VITEK-2 ANC card (BioMérieux, France) and were frozen at − 80℃. The isolates were sub-cultured on blood agar plates, incubated under 35 ℃ for 24 h, and validated by MALDI-TOF MS equipment (micro type MS, Tianrui, China) and 16S rRNA sequencing technology. Epidemiological data for the patients infected with *C. striatum* isolates were collected from the hospital information system.

### Antimicrobial susceptibility test

The antibiotic susceptibility test was performed using the broth microdilution method, and the antibiotics tested include penicillin (0.06–4 μg/mL),cefatriaxone (1–64 μg/mL),

cefepime (1–4 μg/mL), meropenem (0.25–16 μg/mL), gentamicin (4–16 μg/mL), tetracycline (4–16 μg/mL), erythromycin (0.25–8 μg/mL), vancomycin (0.5–4 μg/mL), trimethoprim-sulfamethoxazole (0.5/9.5–4/76 μg/mL), ciprofloxacin (1–4 μg/mL), levofloxacin (2–8 μg/mL), clindamycin (0.25–4 μg/mL), daptomycin (0.5–1 μg/mL), and linezolid (1–2 μg/mL). The antimicrobial susceptibility test and data analysis were performed according to the Clinical and Laboratory Standards Institute guidelines (M45 A2 edition) [10] and the recommendation of the European Committee on Antimicrobial Susceptibility Testing [11].

### Detection of *spa* genes

*C. striatum* isolates were inoculated on the blood agar medium at 35 ℃ for 24 h, and the colonies were picked out and transferred into a 1.5 mL Eppendorf tube with 0.5 mL sterile distilled water. Lysozyme (1 mg/ml) was added into each tube and mixed thoroughly by vortexing, and the tubes were placed into a water bath and incubated at 35 ℃ for 30 min. The total bacterial DNA was extracted according to the instructions of the bacterial genome DNA extraction kit (Beijing Tiangen Company, China). The information on the primers for *spaD*, *spaE*, and *spaF* used in this study is shown in Table 1. The PCR conditions were set as follows: 98 ℃ at 30 s for initial denaturation; 35 cycles including 98 ℃ for 5 s, 56 ℃ for 10 s, and 72 ℃ for 10 s and a final extension at 72 ℃ for 5 min. The primers and PCR conditions were designed in this study, and related *C. striatum* sequences were used as references (Genbank no., CAACYF010000001.1, UFXV01000002.1, and CTEG01000009.1).

**Table 1:** Primer sequences and fragment size of the target genes

Gene	Primer	Sequence (5′ → 3′)	Size (bp)
*spaD*	*spaD*-F *spaD*-R	TCGGTGTTCTTCGGGTATGC CCTGGAAAAGGGTGATTGGAC	267
*spaE*	*spaE*-F *spaE*-R	TTCCTGGTGGCGGTTCCTT CCTGTTCGCGTTCTTTCTCATTAC	241
*spaF*	*spaF*-F *spaF*-R	CATTGTCATTTGCGGATAGGGT CATGGGTGAGGATCTTTCTGGTAC	209

### Pulse-field gel electrophoresis (PFGE)

The optical density of the bacterial cultures of the *C. striatum* isolates tested in this study was adjusted to 3.5–4.0 Mcf and digested with lysostaphin (1 mg/mL) (Merck, USA) at 37 °C for 30 min. The bacterial chromosomal DNA of the isolates was extracted and cleaved using 40 U SwaI (Takara, China). The DNA of the *S. braenderup* H9812 standard strain was extracted and cleaved using 40 U XbaI (Takara, China) and used as the molecular mass standard. Electrophoresis was performed on the CHEF-Mapper XA PFGE system (Bio-Rad, Hercules, CA, USA), and PFGE profiles were analyzed using the Bionumerics v.7.6 software. The isolates with 100% similarity were considered indistinguishable [1, 12], and each genotype was named using a single capital letter.

### Detection of biofilm production

#### Crystal violet staining

For *C. striatum* isolates tested in this study, the bacterial suspensions with turbidity equivalent to 0.5 Mcf were prepared and diluted 100 folds with TSB, and 200 μL of the diluted bacterial suspension was added into the wells in a 96-well polystyrene plate, and incubated at 35℃ for 24 h. The TSB with non-adherent bacteria was removed, and the wells were washed by adding 200 μL PBS. PBS was removed, and the plate was placed at room temperature for 10–20 min to dry. Methanol (200 μl) was added to each well and placed at room temperature for 20 min to dry, and 200 μl of crystal violet solution was added to each well and placed at room temperature for 5–10 min. Then, the plate was gently washed for three times with 200 μL PBS and was placed at room temperature until it was completely dry. Biofilm production was quantified by adding 160 μL glacial acetic acid to destain each well, and the solution was gently mixed for 10 min. The absorbance was measured at 620 nm to quantify the crystal violet present in the destaining solution. Each assay was performed in triplicate. Referring to Brahma U et al. [13], a cut-off value of ODc was set as the mean value of the blank control. The judgment standard was set as follows, OD ≤ ODc, indicating without biofilm formation ability; ODc < OD ≤ 2ODc, indicating weak biofilm formation ability; 2ODc < OD ≤ 4ODc, indicating moderate biofilm formation ability; OD > 4ODc, indicates strong biofilm formation ability.

#### Scanning electron microscopy (SEM)

The *C. striatum* strains with strong and weak biofilm production in the logarithmic growth period were transferred to a 6-well plate containing TSB liquid medium (the initial amount of bacteria in the well was 10^7^ CFU). A sterile plastic coverslip (Thermanox™) was put into each well to collect the biofilm and incubate it aerobically at 35 ℃ for 24 h. After removing the culture medium, the coverslip was fixed in 0.1 M cacodylate sodium buffer (pH 7.2) with 2.5% glutaraldehyde at room temperature for 30 min, and in the same buffer with 1%OsO_4_ solution containing 2.5 mM CaCl_2_ at room temperature for 30 min. The biofilm was dehydrated in ascending acetone series and dried. Subsequently, the samples were observed with scanning electron microscope after drying [14].

#### Biofilm degradation assay

The degrading activities of three kinds of well-known extracellular matrix degradative agents on the biofilm of *C. striatum* were investigated in this study, including protein degrading enzyme (proteinase K), polysaccharide poly-N-acetylglucosamine (PNAG): degrading enzyme (dispersin B), and eDNA degrading enzyme (DNase I). The assays were performed according to the protocols recommended previously [9, 15]. Proteinase K (RT403, 20 mg/mL) was purchased from TIANGEN Biotech (Beijing) Co., Ltd. β-N-Acetylglucosaminidase (S10237, 69.3 U/mL) was purchased from Shanghai yuanye Bio-Technology Co., Ltd, and DNase I (D8071, 5 mg/ml) from Beijing Solarbio Science & Technology Co., Ltd. Biofilm formation assay was performed as above mentioned. After 24 h of incubation, the biofilm was washed with 200 μL PBS three times. Then, 200 μL of the degrading agent was carefully added to the wells with biofilm and incubated at 37 ℃ for 2 h. The wells were washed with PBS for three times and the biofilm was stained with crystal violet. The following groups were designed to observe the biofilm degrading effects of different kinds of the agents, including control group (without degrading agents), single degrading agent groups (20 μg/mL proteinase K, 20 μg/mL dispersin B, and 20 μg/mL DNase I), combination groups of degrading agents (20 μg/mL proteinase K, and 20 μg/mL dispersin B; 20 μg/mL proteinase K, and DNase I; 20 μg/mL dispersin B, and DNase I). All the tests were performed in triplicate. The data were analyzed with an independent-samples t-test using IBM SPSS Statistics 23.0 software, and *p* < 0.05 was deemed statistically significant.

## Results

Epidemiological data showed that the average age of twenty-seven patients infected with *C. striatum* was 54.63-years-old, and the male/female ratio was 22:5. The average time from admission to the isolation of *C. striatum* isolates was 24.04 days. Around 96.30% (26/27) of the patients received antimicrobial treatment two weeks before *C. striatum* isolation. The most frequently prescribed antimicrobials are β-lactam antibiotic/β-lactamase inhibitor combinations (66.67%, 18/27), followed by fluoroquinolones (44.44%, 12/27), carbapenems, (44.44%, 12/27) and cephalosporins (14.81%, 4/27). Two patients died during hospitalization, and the remaining patients were discharged after recovery. The detailed epidemiological data are summarized in Additional file 1: Table S1.

As shown in Table 2, twenty-seven clinical *C. striatum* isolates were all sensitive to vancomycin, linezolid, daptomycin, and the resistance rate to gentamicin was lower (7.41%). However, the resistance rates to penicillin, cefepime, meropenem, tetracycline and erythromycin were high (100%, 92.59%, 85.19%, 81.48%, and 88.89%), while the resistance rates to penicillin and ceftriaxone were the highest, reaching up to 100% (27/27). Around 96.3% (26/27) of *C. striatum* isolates were deemed multidrug-resistant (MDR) in at least three different kinds of the antimicrobials tested in this study. CS-14 isolate was the only non-MDR isolate resistant to penicillin and ceftriaxone.

**Table 2:** Results of in vitro antimicrobial susceptibility test of 27 *C. striatum* isolates

Strains	PEN	CRO	FEP	MEM	GEN	TCY	ERY	VAN	SXT	CIP	CLI	DAP	LNZ	MDR
CS-1	R	R	R	R	S	R	R	S	R	R	R	S	S	+
CS-2	R	R	R	R	S	R	R	S	R	R	R	S	S	+
CS-5	R	R	R	R	S	S	R	S	S	R	R	S	S	+
CS-9	R	R	R	R	S	R	R	S	R	R	R	S	S	+
CS-11	R	R	R	R	S	R	R	S	R	R	R	S	S	+
CS-14	R	R	I	S	S	S	S	S	S	S	I	S	S	-
CS-17	R	R	R	R	S	R	R	S	R	R	R	S	S	+
CS-20	R	R	R	I	S	R	R	S	R	R	R	S	S	+
CS-30	R	R	R	R	S	R	R	S	R	R	R	S	S	+
CS-32	R	R	R	R	S	R	R	S	R	R	R	S	S	+
CS-36	R	R	R	R	S	R	R	S	R	R	R	S	S	+
CS-51	R	R	R	I	R	R	R	S	R	R	R	S	S	+
CS-176	R	R	R	R	S	R	R	S	S	R	R	S	S	+
CS-177	R	R	I	S	S	S	R	S	S	R	R	S	S	+
CS-178	R	R	R	R	I	S	R	S	R	R	R	S	S	+
CS-179	R	R	R	R	S	R	R	S	R	R	R	S	S	+
CS-180	R	R	R	R	I	S	I	S	R	R	R	S	S	+
CS-250	R	R	R	R	S	R	R	S	R	R	R	S	S	+
CS-251	R	R	R	R	S	R	R	S	R	R	R	S	S	+
CS-252	R	R	R	R	S	R	R	S	R	R	R	S	S	+
CS-253	R	R	R	R	S	R	R	S	R	R	R	S	S	+
CS-254	R	R	R	R	S	R	R	S	R	R	R	S	S	+
CS-255	R	R	R	R	S	R	R	S	R	R	R	S	S	+
CS-256	R	R	R	R	S	R	R	S	R	R	R	S	S	+
CS-257	R	R	R	R	S	R	R	S	R	R	R	S	S	+
CS-258	R	R	R	R	S	R	S	S	R	R	I	S	S	+
CS-259	R	R	R	R	R	R	R	S	R	R	R	S	S	+

R, resistant; I, intermediate; S, sensitive

*PEN* penicillin, *CRO* cefatriaxone, *FEP* cefepime, *MEM* meropenem, *GEN* gentamicin, *TCY* tetracycline, *ERY* erythromycin, *VAN* vancomycin, *SXT* trimethoprim-sulfamethoxazole, *CIP* ciprofloxacin, *CLI* clindamycin, *DAP* daptomycin, *LNZ* linezolid, *MDR* multi-drug resistance

The PCR results revealed that the positive rates of *spaD*, *spaE* and *spaF* genes were 81.48% (22/27), 85.19% (23/27), and 85.19% (23/27), respectively. Around 74.07% (20/27) of the *C. striatum* isolates carried *spaD*, *spaE* and *spaF* genes concurrently, while 7.41% (2/27) of *C. striatum* isolates did not carry any of *spaD*, *spaE* or *spaF* genes. Around 11.11% (3/27) of the isolates were positive with *spaD* and *spaF genes*, but the *spaE* gene was not detected. In addition, 7.41% (2/27) of the *C. striatum* isolates only carried the *spaD* gene.

**Fig.1:**
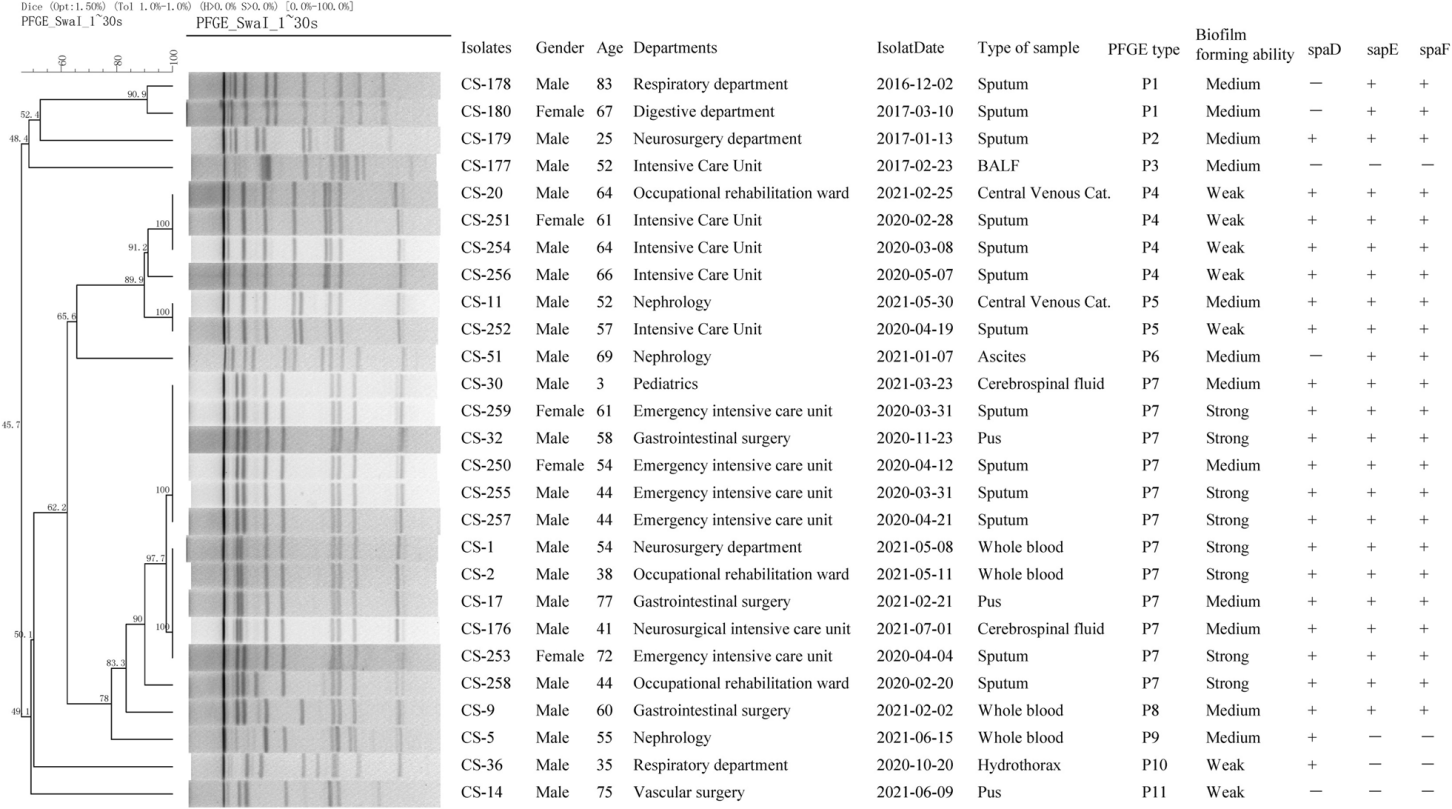
PFGE analysis of 27 *C. Striatum* isolates

Based on the discrimination standard for PFGE genotyping proposed by Tenover et al.[12], twenty-seven *C. striatum* isolates were divided into eleven genotypes (P1 ~ P11), the P7 is the predominant genotype and accounted for 44.44% (12/27), as shown in Fig. 1. The P7 genotype contained twelve strains of *C. striatum* isolated from 31st, 2020 to 11th, 2021. Half of the samples were isolated from the intensive care unit (ICU), and 50% (6/12) of the specimens were isolated from qualified sputum samples. Six isolates were isolated from the cerebrospinal fluid (16.67%, 2/12), pus (16.67%, 2/12), and whole blood (16.67%, 2/12), respectively. In addition, most of the strains belonging to the P7 genotype presented stronger biofilm formation abilities and were all multidrug-resistant (MDR). Even worse, four isolates belonging to the P7 genotype were sequentially isolated from the patients admitted to ICU between March 31st and April 21st, 2020, indicating a potential nosocomial outbreak. Also, the isolates belonging to another genotype (P4) with weak biofilm formation ability accounted for 14.8% (4/27), three of which were collectively isolated from the patients admitted to ICU from February 8th to May 5th, 2020, which indicated a possible nosocomial transmission.

**Fig. 2:**
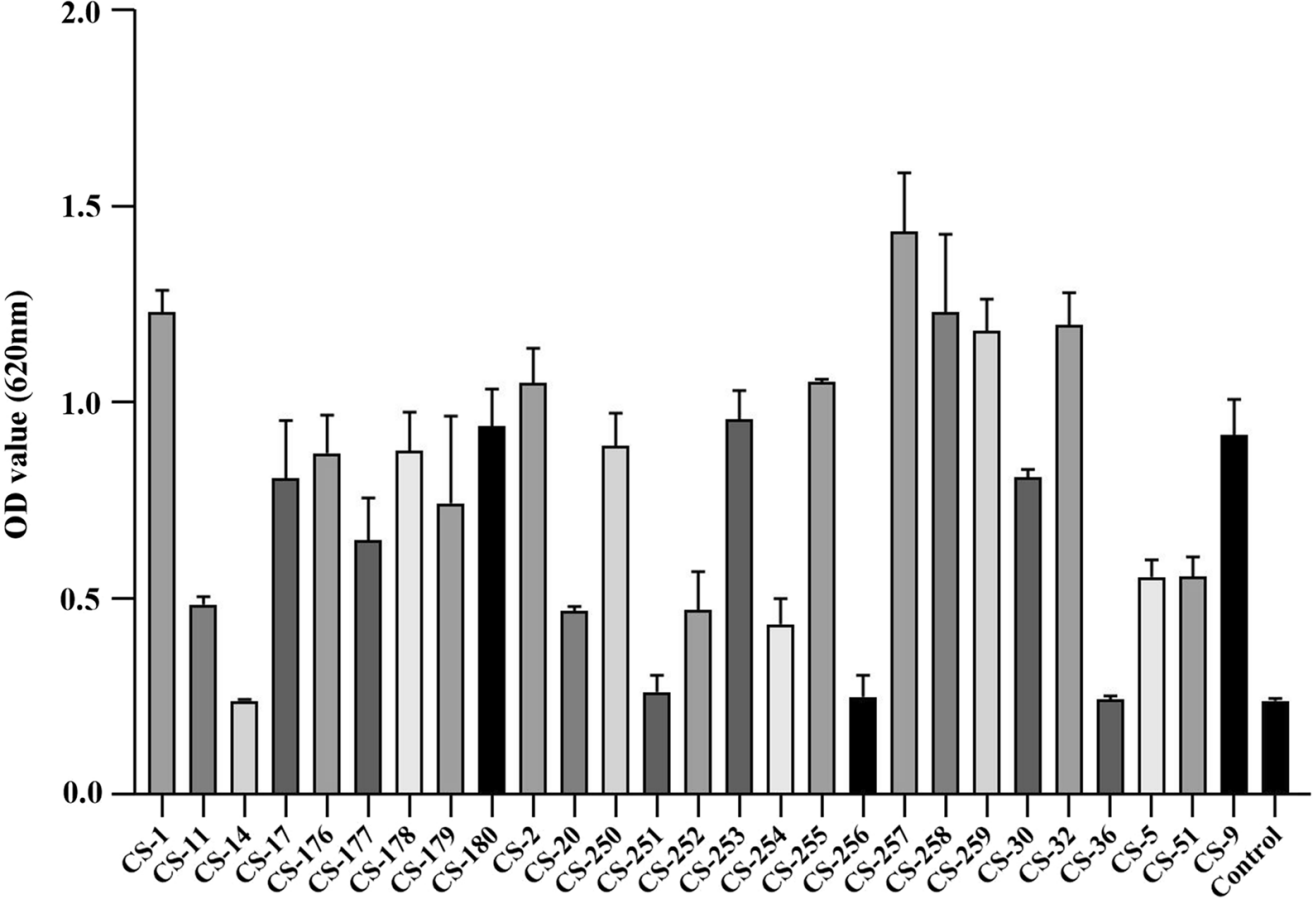
Biofilm forming abilities of 27 *C. striatum*

**Fig. 3:**
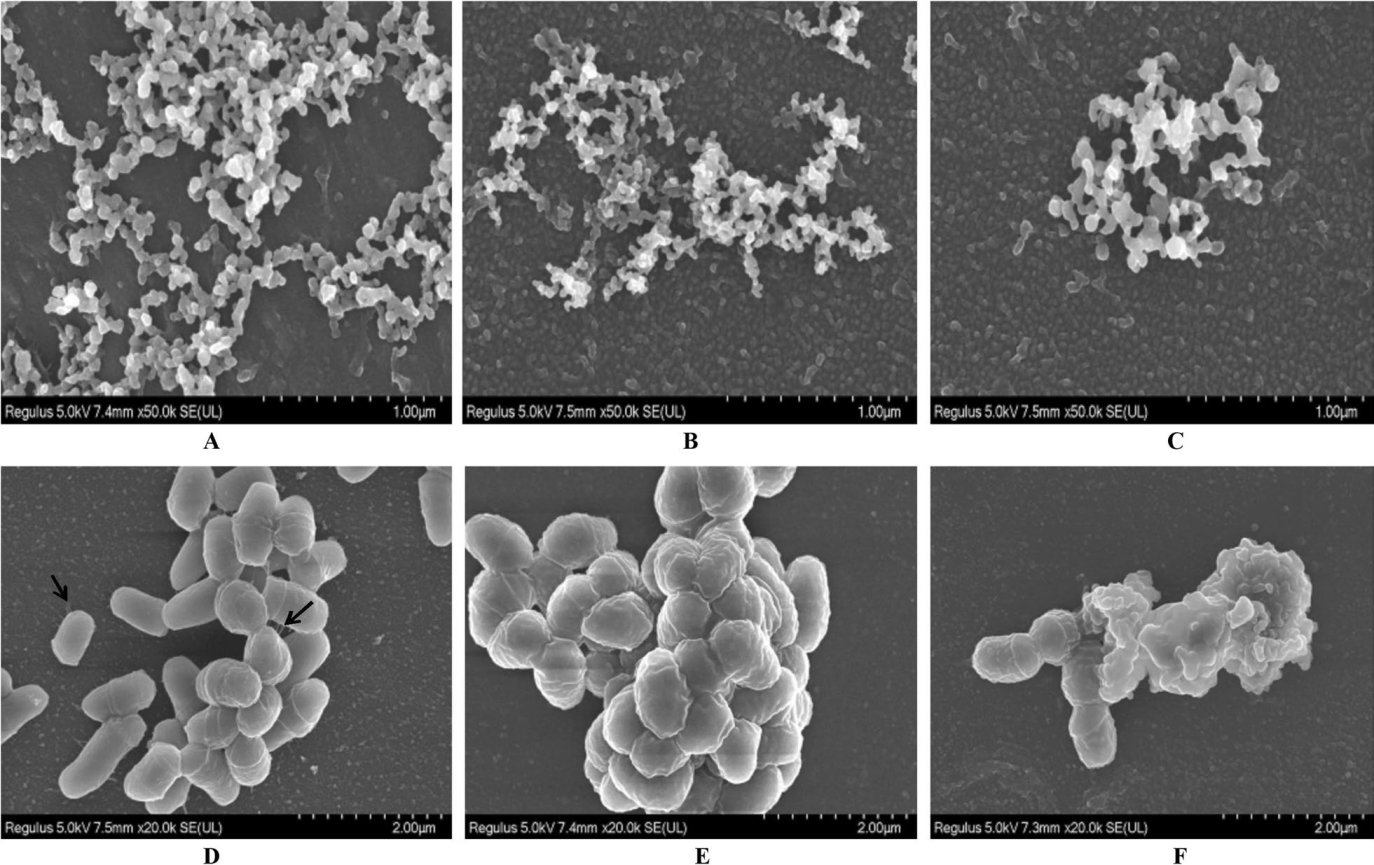
Morphological features of *C. striatum* strains strong and weak biofilm formation abilities. **A**, **D**. *C. striatum* isolate with strong biofilm formation ability; **B**, **E**. *C. striatum* isolate with medium biofilm forming ability. **C**, **F**. *C. striatum* isolate with weak biofilm forming ability. Black arrow (→) indicates the filaments produced by the *C. striatum* isolate with strong biofilm formation ability

As shown in Fig. 2, twenty-seven *C. striatum* strains were all biofilm producers but the biofilm production abilities differed significantly. Eight isolates were potent biofilm producers (29.63%, 8/27), twelve were medium biofilm producers (44.44%, 12/27), while the remaining seven isolates showed weak biofilm production abilities (25.93%, 7/27). Most isolates (51.85%, 14/27) were isolated from the lower respiratory tract samples and all the strong biofilm producers belonged to the P7 genotype. The SEM results confirmed the presence of mature and high-density of biofilm in the strong biofilm producers (Fig. 3), compared with those with weak biofilm production abilities.

**Fig. 4:**
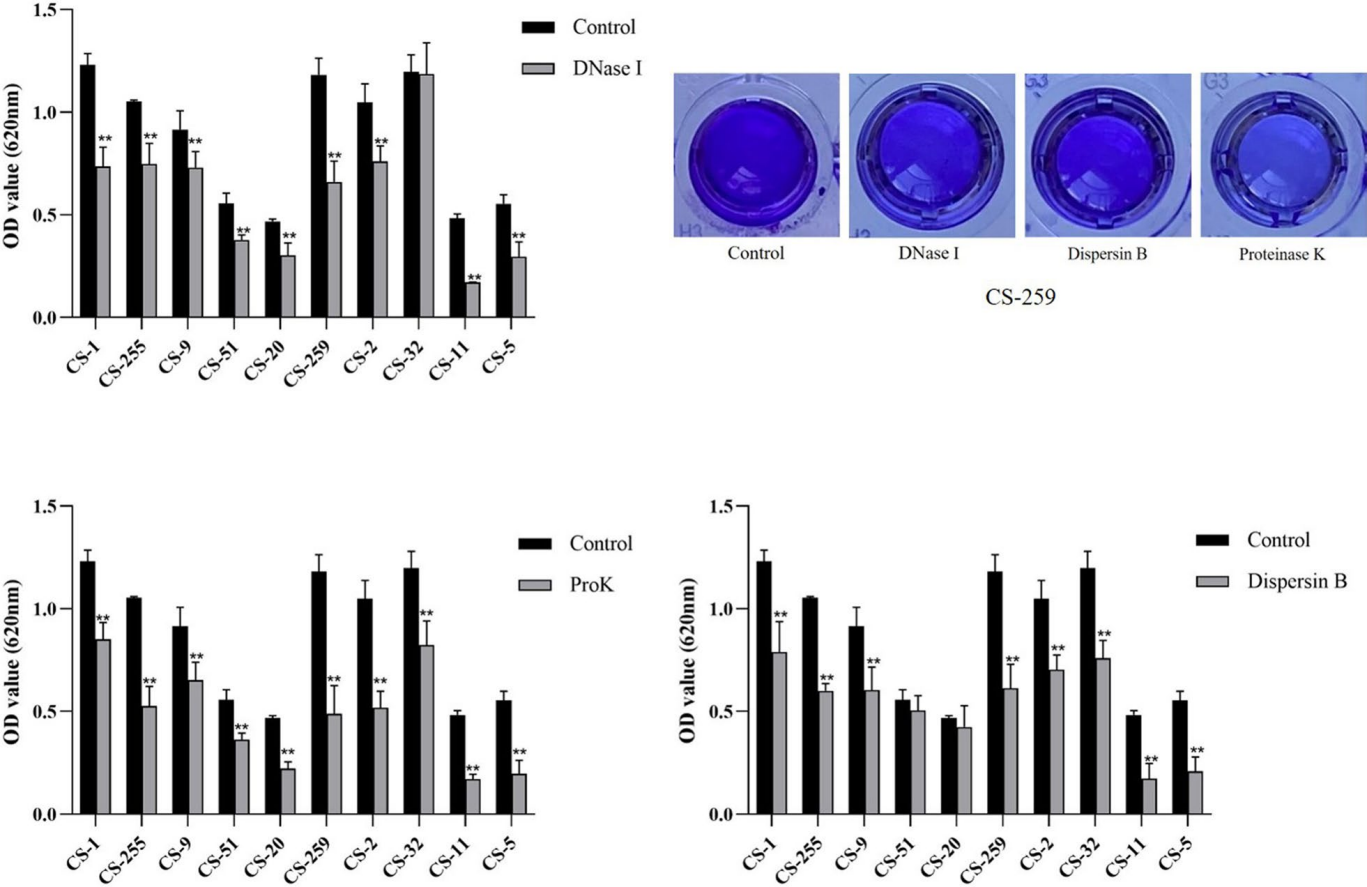
Biofilm eliminating effects of biofilm degrading agents (proteinase K, dispersin B and DNase I)

**Fig.5:**
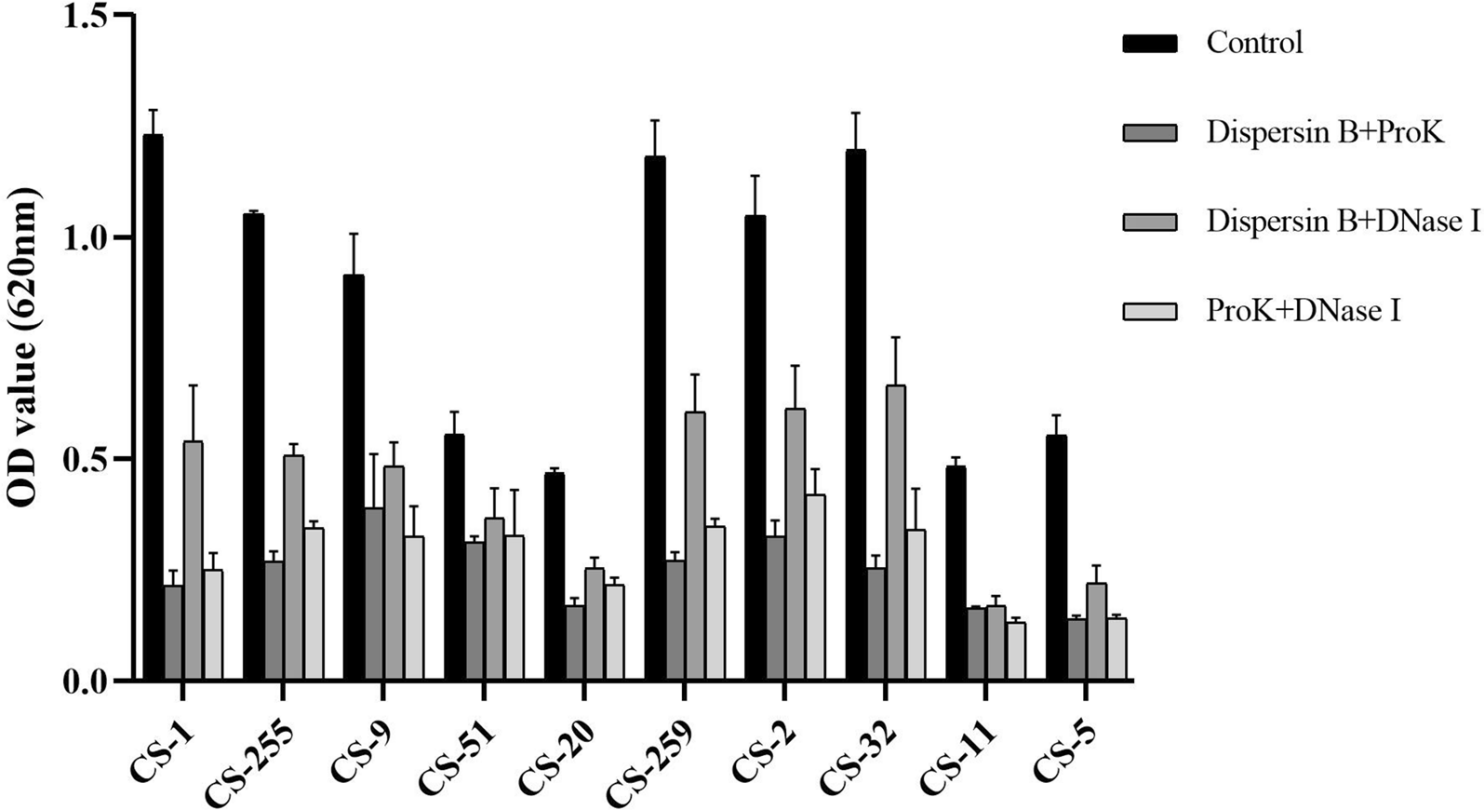
Biofilm eliminating effects of combinations of biofilm degrading agents (proteinase K, dispersin B and DNase I)

As shown in Fig. 4, the biofilm of 80% (8/10), 90% (9/10), and 100% (10/10) *C. striatum* isolates were significantly eliminated upon the treatment with 20 μg/mL dispersin B, DNase I, and proteinase K (*p* < 0.05), respectively. The degradation efficiency of the above three agents differed significantly, when proteinase K showed the most potent degrading effects, followed by dispersin B and DNase I (Fig. 4). The degrading percentages of the biofilm of 50% (5/10) isolates (CS-20, CS-259, CS-2, CS-11, and CS-5) exceeded 50% when treated with proteinase K, while only 20% (2/10) and 10% (1/10) for dispersin B and DNase I groups. For combination groups, the combination of proteinase K/dispersin B showed the most potent biofilm-eliminating effects, followed by the combination of proteinase K/eDNA and dispersin B/DNase I (Fig. 5 and Additional file 2: Table S2). The degrading percentages of the biofilm of 90% (9/10) isolates exceeded 50% when treated with combination of proteinase K/dispersin B, followed by combination of proteinase K/DNase I (80%,8/10) and dispersin B/DNase I (40%, 4/10). However, the degrading percentage of the biofilm of CS-51 isolate did not exceed 50% when treated with any combinations of the degrading agents.

## Discussion

In this study, almost all of the *C. striatum* strains investigated in this study were multidrug -resistant. They were only sensitive to vancomycin, linezolid and daptomycin, consistent with some previous global studies [5, 16, 17]. *C. striatum* isolates were mainly isolated from lower respiratory tract specimens (51.85%, 14/27). A dominant genotype, P7, was identified during this study, triggering a nosocomial outbreak among ICU patients between March and April, 2020. Furthermore, all of the isolates belonging to P7 genotype were confirmed to be medium or strong biofilm producers and multidrug-resistant. Rapid and accurate discrimination of predominant *C. striatum* clones may help clinicians implement more potent measures to control its nosocomial transmission or treat of infections caused by *C. striatum*.

Some previous studies revealed that MDR *C. striatum* could produce mature biofilms on several kinds of surfaces, such as medical catheters [3], leading to chronic infections. Even worse, a recent report revealed significant resistance of some dominant *C. striatum* clones to some widely used biocides (such as glutaraldehyde), especially for biofilm-associated forms of *C. striatum* [18]. Exploration of new kinds of anti-biofilm agents for biofilm-producing *C. striatum* seems to be urgent. In this study, all *C. striatum* isolates were confirmed to be biofilm producers, and 74.1% (20/27) isolates were confirmed to have medium and strong biofilm producing abilities. Also, the isolates belonging to the same PFGE type or closely similar types presented identical or similar biofilm formation abilities. However, the consistency between resistance features and clonality of the isolates was not observed, which is inconsistent with another previous study [4].

Biofilm formation ability is essential for some bacterial pathogens [19]. Cellular surface components (such as pili or fimbriae [6]) and extracellular matrix [7] are two critical determinants in some well-known pathogens. Fouzia Nasim et al. [20] and Sana Alibi et al. [6] demonstrated that the adhesion ability in *Corynebacterium spp.* was generally mediated by fimbriae, which is usually encoded by three separate fimbriae gene clusters, *spaDEF* gene. Also, *C. striatum* fimbriae can also mediate its specific adhesion to host cells. Sana Alibi et al. revealed that *Spa-type* genes were detected in all *C. striatum* strains. However, not all the *C. striatum* strains (74.07%, 20/27) carried *spaD*, *spaE,* and *spaF* genes in this study. In addition, eight *C. striatum* strains with strong biofilm-forming ability carried *spaDEF*. At the same time, five *C. striatum* strains with weak biofilm-forming ability were also positive with *spaDEF* genes, which indicates an indefinite relationship between biofilm formation abilities and expression of pili in *C. striatum*. For some well-known gram-positive bacteria (including *S. aureus*, and *Bacillus subtilis*), the bacterial extracellular matrix has been recognized to be an essential determinant in mediating biofilm formation and participating in the protection process from external insults [21, 22]. The extracellular matrix composition was similar among different bacteria, including EPA, proteins, and eDNA, all of which participated in the process of biofilm formation in different manners [21, 23–25]. Correspondingly, degradative agents directed against the above components were developed to deter biofilm formation, including dispersin B, proteinase K, and DNase I. Biofilm matrix degradation with enzymatic degradative agents toward polysaccharides, proteins, and nucleic acids paves an efficient way of controlling or treating infections caused by biofilm-producing bacterial pathogens [26–28]. A previous report revealed that adding exogenous DNA would promote biofilm formation in *Corynebacterium glutamicum*, which suggests a nucleic acids’ potential role in regulating biofilm formation in *Corynebacterium* spp. [29]. Nevertheless, to the best of our knowledge, the actual composition of the extracellular matrix of *C. striatum* and the potential roles of biofilm matrix degradative agents was not reported yet.

In this study, biofilm-degradative agents (dispersin B, DNase I, and proteinase K) were employed in *C. striatum* biofilm degrading assays, all of which were commonly used in the assays for some other well-known bacterial pathogens, such as *S. aureus* [9, 15]. Interestingly, significant degradative effects of the above biofilm-degradative agents were observed. Overall, proteinase K showed the most potent biofilm degradative effects. The most effective biofilm-eliminating effect was observed in proteinase K and dispersin B or DNase I groups. Meanwhile, significant diversity of biofilm-eliminating effects of biofilm matrix degradative agents on *C. striatum* biofilm was observed during this study, which suggests a significant strain or clonal heterogenicity of the composition of extracellular matrix in *C. striatum*. Specific constituents of the extracellular matrix in determining the biofilm formation capability of *C. striatum* and possible molecular mechanisms need to be further investigated, especially for extracellular proteins, which may be potent anti-biofilm targets.

## Conclusions

Multidrug-resistant *C. striatum* isolates accounted for the most of the isolates investigated in this study. The predominant clone identified in this study presented a more potent biofilm formation ability, and a nosocomial outbreak was observed. Also, this study firstly revealed the potential roles of the extracellular matrix in mediating *C. striatum* biofilm formation, since significant biofilm-eliminating effects of extracellular matrix degradative agents toward *C. striatum* were observed, especially for protein degradative agents. Further exploration of the extracellular matrix composition of *C. striatum* and a deep understanding of the molecular mechanism of extracellular matrix in mediating the biofilm formation of *C. striatum* will pave a new way for us to control and treat of colonization or infections caused by biofilm-producing *C. striatum* strains.

## Supplementary Information

**Additional file 1: Table S1.** Epidemiology of twenty-seven patients infected with multi-drug resistant *Corynebacterium striatum*.

**Additional file 2: Table S2.** The results of statistical analysis of the biofilm eliminating effects of combinations of biofilm degrading agents (proteinase K, dispersin B and DNase I).

## Data Availability

None.

## Abbreviations

PFGE: Pulsed-field gel electrophoresis
PCR: Polymerase chain reaction
MIC: Minimum inhibitory concentration
ICU: Intensive care unit
MDR: Multi-drug resistant
SEM: Scanning electron microscope
EPS: Exopolysaccharides
eDNA: Extracellular DNA
