# Supplementary material for: Antibioflm effects of extracellular matrix degradative agents on the biofilm of different strains of multi-drug resistant Corynebacterium striatum
Source: Ann Clin Microbiol Antimicrob. 2022 Nov 25;21:53. doi: 10.1186/s12941-022-00546-y (PMC9700914; doi:10.1186/s12941-022-00546-y)
Supplement: Supplementary file 1 — Additional file 1: Table S1. Epidemiology of twenty-seven patients infected with multi-drug resistant Corynebacterium striatum. [file 12941_2022_546_MOESM1_ESM.doc]

# Supplementary table 1 Epidemiology of twenty-seven patients infected with multi-drug resistant *Corynebacterium striatum*

| **No. of Isolates** | **Department** | **Age** | **Gender** | **Clinical diagnosis** | **Usage of antimicrobial agents two weeks ago before *C. striatum* isolation** | **Outcome** | **Date of hospitalization** | **Date of *C. striatum* isolation** |
| --- | --- | --- | --- | --- | --- | --- | --- | --- |
| CS-1 | Neurosurgery department | 54 | Male | Traumatic intracranial hemorrhage, Hypertension | β-lactam antibiotic/β-lactamase inhibitor combinations，carbapenem | Discharge | 2021-04-12 | 2021-05-08 |
| CS-2 | Occupational rehabilitation ward | 38 | Male | Recovery period of cerebral hemorrhage | Fluoroquinolones，β-lactam antibiotic/β-lactamase inhibitor combinations | Discharge | 2021-04-09 | 2021-05-11 |
| CS-5 | Nephrology | 55 | Male | Chronic nephrosis | Fluoroquinolones，Cephalosporins | Discharge | 2021-05-25 | 2021-06-15 |
| CS-9 | Gastrointestinal surgery | 60 | Male | Gastric cancer, Hypertension, Miocardial infarction | Fluoroquinolones，carbapenem | Discharge | 2021-01-14 | 2021-02-02 |
| CS-11 | Nephrology | 52 | Male | Chronic nephrosis, Type II Diabetes | Fluoroquinolones | Discharge | 2021-05-18 | 2021-05-30 |
| CS-14 | Vascular surgery | 75 | Male | Diabetic foot, Type II diabetes | Fluoroquinolones，β-lactam antibiotic/β-lactamase inhibitor combinations | Discharge | 2021-04-17 | 2021-06-09 |
| CS-17 | Gastrointestinal surgery | 77 | Male | Rectal cancer, Hypertension、COPD | Carbapenem，β-lactam antibiotic/β-lactamase inhibitor combinations | Discharge | 2021-01-06 | 2021-02-21 |
| CS-20 | Occupational rehabilitation ward | 64 | Male | Recovery period of brain trauma | Carbapenem，Fluoroquinolones，β-lactam antibiotic/β-lactamase inhibitor combinations | Discharge | 2021-02-15 | 2021-02-25 |
| CS-30 | Pediatrics | 3 | Male | Epilepsy | None | Discharge | 2021-03-18 | 2021-03-23 |
| CS-32 | Gastrointestinal surgery | 58 | Male | Upper gastrointestinal bleeding, Gastric cancer | β-lactam antibiotic/β-lactamase inhibitor combinations | Discharge | 2020-10-29 | 2020-11-23 |
| CS-36 | Respiratory department | 35 | Male | Pulmonary infection | β-lactam antibiotic/β-lactamase inhibitor combinations，Fluoroquinolones， | Discharge | 2020-10-14 | 2020-10-20 |
| CS-51 | Nephrology | 69 | Male | Inguinal hernia incarceration, Hypertension、Uremia | Fluoroquinolones，carbapenem | Discharge | 2020-12-16 | 2021-01-07 |
| CS-176 | Neurosurgical intensive care unit | 41 | Male | Hypertensive cerebral hemorrhage, type II diabetes | Fluoroquinolones，β-lactam antibiotic/β-lactamase inhibitor combinations | Discharge | 2021-06-17 | 2021-07-01 |
| CS-177（C339） | Intensive Care Unit | 52 | Male | Recovery period of brain trauma | β-lactam antibiotic/β-lactamase inhibitor combinations | Discharge | 2017-02-23 | 2017-02-23 |
| CS-178（C453） | Respiratory department | 83 | Male | Pulmonary infection, pulmonary tumor | Carbapenem，Cephalosporins | Death | 2016-08-22 | 2016-12-02 |
| CS-179（C472） | Neurosurgery department | 25 | Male | Traumatic intracranial hemorrhage | β-lactam antibiotic/β-lactamase inhibitor combinations | Discharge | 2016-12-20 | 2017-01-13 |
| CS-180（C476） | Digestive department | 67 | Female | Upper gastrointestinal bleeding, Gastric cancer | Carbapenem，Fluoroquinolones | Discharge | 2017-03-06 | 2017-03-10 |
| CS-250 | Emergency intensive care unit | 54 | Female | Severe pneumonia, Hypertension | Carbapenem，Fluoroquinolones，Cephalosporins | Discharge | 2020-03-22 | 2020-04-12 |
| CS-251 | Intensive Care Unit | 61 | Female | Coma, type II Diabetes | β-lactam antibiotic/β-lactamase inhibitor combinations | Discharge | 2020-02-17 | 2020-02-28 |
| CS-252 | Intensive Care Unit | 57 | Male | Pelvic fracture | β-lactam antibiotic/β-lactamase inhibitor combinations | Death | 2020-04-11 | 2020-04-19 |
| CS-253 | Emergency intensive care unit | 72 | Female | Septic shock, Hypertension, Rheumatoid arthritis | Carbapenem | Discharge | 2020-03-27 | 2020-04-04 |
| CS-254 | Intensive Care Unit | 64 | Male | Liver failure, Hypertension | β-lactam antibiotic/β-lactamase inhibitor combinations | Discharge | 2020-02-14 | 2020-03-08 |
| CS-255 | Emergency intensive care unit | 44 | Male | Traumatic intracranial hemorrhage | β-lactam antibiotic/β-lactamase inhibitor combinations | Discharge | 2020-03-19 | 2020-03-31 |
| CS-256 | Intensive Care Unit | 66 | Male | Septic shock | β-lactam antibiotic/β-lactamase inhibitor combinations，Cephalosporins，Carbapenem | Discharge | 2020-04-29 | 2020-05-07 |
| CS-257 | Emergency intensive care unit | 44 | Male | Consciousness disorder | β-lactam antibiotic/β-lactamase inhibitor combinations | Discharge | 2020-03-11 | 2020-04-21 |
| CS-258 | Occupational rehabilitation ward | 44 | Male | Recovery period of cerebral hemorrhage | β-lactam antibiotic/β-lactamase inhibitor combinations，Carbapenem | Discharge | 2019-12-02 | 2020-02-20 |
| CS-259 | Emergency intensive care unit | 61 | Female | Pneumonia, Connective tissue disease | Carbapenem，Fluoroquinolones，β-lactam antibiotic/β-lactamase inhibitor combinations | Discharge | 2020-03-13 | 2020-03-31 |
