# Supplementary material for: Antibioflm effects of extracellular matrix degradative agents on the biofilm of different strains of multi-drug resistant Corynebacterium striatum
Source: Ann Clin Microbiol Antimicrob. 2022 Nov 25;21:53. doi: 10.1186/s12941-022-00546-y (PMC9700914; doi:10.1186/s12941-022-00546-y)
Supplement: Supplementary file 2 — Additional file 2: Table S2. The results of statistical analysis of the biofilm eliminating effects of combinations of biofilm degrading agents (proteinase K, dispersin B and DNase I). [file 12941_2022_546_MOESM2_ESM.docx]

**Supplementary Table 2**

| Groups | t | *P* |
| --- | --- | --- |
| **CS-1** | | |
| Control vs DspB&ProK | 26.789 | 0.000 |
| Control vs DspB&DNase I | 8.648 | 0.001 |
| Control vs ProK&DNase I | 25.125 | 0.000 |
| DspB&ProK vs DspB&DNase I | -4.275 | 0.013 |
| DspB&ProK vs ProK&DNase I | -1.170 | 0.307 |
| DspB&DNase I vs ProK&DNase I | 3.785 | 0.019 |
| **CS-255** | | |
| Control vs DspB&ProK | 55.132 | 0.000 |
| Control vs DspB&DNase I | 35.212 | 0.000 |
| Control vs ProK&DNase I | 72.176 | 0.000 |
| DspB&ProK vs DspB&DNase I | -11.772 | 0.000 |
| DspB&ProK vs ProK&DNase I | -4.632 | 0.01 |
| DspB&DNase I vs ProK&DNase I | 9.315 | 0.001 |
| **CS-9** | | |
| Control vs DspB&ProK | 6.003 | 0.004 |
| Control vs DspB&DNase I | 7.039 | 0.002 |
| Control vs ProK&DNase I | 8.945 | 0.001 |
| DspB&ProK vs DspB&DNase I | -1.214 | 0.292 |
| DspB&ProK vs ProK&DNase I | 0.089 | 0.464 |
| DspB&DNase I vs ProK&DNase I | 3.117 | 0.036 |
| **CS-51** | | |
| Control vs DspB&ProK | 8.123 | 0.001 |
| Control vs DspB&DNase I | 3.931 | 0.017 |
| Control vs ProK&DNase I | 3.440 | 0.026 |
| DspB&ProK vs DspB&DNase I | -1.411 | 0.231 |
| DspB&ProK vs ProK&DNase I | -0.237 | 0.834 |
| DspB&DNase I vs ProK&DNase I | 0.573 | 0.598 |
| **CS-20** | | |
| Control vs DspB&ProK | 24.551 | 0.000 |
| Control vs DspB&DNase I | 13.490 | 0.000 |
| Control vs ProK&DNase I | 22.060 | 0.000 |
| DspB&ProK vs DspB&DNase I | -4.743 | 0.009 |
| DspB&ProK vs ProK&DNase I | -3.454 | 0.026 |
| DspB&DNase I vs ProK&DNase I | 2.123 | 0.113 |
| **CS-259** | | |
| Control vs DspB&ProK | 14.858 | 0.001 |
| Control vs DspB&DNase I | 8.469 | 0.001 |
| Control vs ProK&DNase I | 17.404 | 0.002 |
| DspB&ProK vs DspB&DNase I | -5.157 | 0.014 |
| DspB&ProK vs ProK&DNase I | -4.664 | 0.019 |
| DspB&DNase I vs ProK&DNase I | 5.082 | 0.031 |
| **CS-2** | | |
| Control vs DspB&ProK | 12.969 | 0.000 |
| Control vs DspB&DNase I | 5.708 | 0.005 |
| Control vs ProK&DNase I | 10.286 | 0.001 |
| DspB&ProK vs DspB&DNase I | -4.821 | 0.009 |
| DspB&ProK vs ProK&DNase I | -2.450 | 0.070 |
| DspB&DNase I vs ProK&DNase I | 2.983 | 0.041 |
| **CS-32** | | |
| Control vs DspB&ProK | 18.486 | 0.000 |
| Control vs DspB&DNase I | 6.727 | 0.003 |
| Control vs ProK&DNase I | 11.878 | 0.000 |
| DspB&ProK vs DspB&DNase I | -6.367 | 0.003 |
| DspB&ProK vs ProK&DNase I | -1.529 | 0.201 |
| DspB&DNase I vs ProK&DNase I | 3.956 | 0.017 |
| **CS-11** | | |
| Control vs DspB&ProK | 28.355 | 0.000 |
| Control vs DspB&DNase I | 16.330 | 0.000 |
| Control vs ProK&DNase I | 25.466 | 0.000 |
| DspB&ProK vs DspB&DNase I | -0.481 | 0.656 |
| DspB&ProK vs ProK&DNase I | 4.657 | 0.010 |
| DspB&DNase I vs ProK&DNase I | 2.796 | 0.049 |
| **CS-5** | | |
| Control vs DspB&ProK | 15.907 | 0.000 |
| Control vs DspB&DNase I | 9.597 | 0.001 |
| Control vs ProK&DNase I | 15.765 | 0.000 |
| DspB&ProK vs DspB&DNase I | -3.345 | 0.072 |
| DspB&ProK vs ProK&DNase I | -0.146 | 0.891 |
| DspB&DNase I vs ProK&DNase I | 3.277 | 0.072 |

Note: DspB:dispersin B; ProK:proteinase K
